# Number of Days Required to Estimate Habitual Vegetable Variety: A Cross-Sectional Analysis Using Dietary Records for 7 Consecutive Days

**DOI:** 10.3390/nu14010056

**Published:** 2021-12-24

**Authors:** Ryoko Kurisaki, Osamu Kushida

**Affiliations:** Department of Nutrition and Life Sciences, School of Food and Nutritional Sciences, University of Shizuoka, 52-1 Yada, Suruga-ku, Shizuoka 422-8526, Japan; f17111@u-shizuoka-ken.ac.jp

**Keywords:** vegetable, variety, habitual, dietary records, capture proportion

## Abstract

The aim of this cross-sectional study was to examine the number of days required to estimate habitual vegetable variety by conducting a multiday, dietary record. Sixty respondents from three groups in Japan (rural residents, general students, and nutrition students) participated in the study using a self-administered questionnaire in September 2018. To measure vegetable variety, the number of different vegetables consumed was extracted from the dietary records of seven consecutive days. Differences in the number of vegetables consumed and the capture proportion over seven consecutive days between groups were examined using repeated measures analysis of variance and one-way analysis of variance. The vegetable variety between each day was also compared using Pearson’s correlation coefficient. The vegetable variety based on dietary records for seven consecutive days confirmed the differences between groups by repeated measurements (*p* = 0.013). However, there was no significant difference among groups in the capture proportion per survey day based on seven consecutive days. Furthermore, there were significant correlations between the number of vegetables consumed over seven consecutive days and that consumed on two or more days (r > 0.50, *p* < 0.01) and especially three or more days in all groups (r > 0.70, *p* < 0.001). The present study suggested that a dietary survey over two or more days could provide an estimate of habitual vegetable variety.

## 1. Introduction

The association of the amount of fruits and vegetables (FV) consumed with chronic diseases such as cardiovascular disease and cancer has been analyzed by many nutritional epidemiological studies [1,2,3]. Because inadequate intake of FV contributes to many chronic diseases and global excess mortality, the World Health Organization (WHO) recommends increasing the amount of FV consumed globally [4].

Recently, many studies have examined the association between health status and FV variety rather than consumed amount of FV. For example, vegetable variety has been reported to be positively correlated with overall diet quality [5] and higher cognitive function [6]. Vegetable variety has also been reported to reduce the onset risk of chronic diseases such as type 2 diabetes [7] and lung cancer [8]. Regarding cognitive function [6] and lung cancer [8], because vegetable variety has been more strongly associated with health status than the consumed amount of vegetables, it is important to increase vegetable variety. The American and Australian dietary guidelines also make recommendations to increase the vegetable variety in subgroups, not just the consumed amount [9,10].

According to a scoping review summarizing the operationalization of FV variety [11], many studies have used a food frequency questionnaire (FFQ) or 24-h dietary recall (24-h recall) to measure FV variety. In general, an FFQ is intended to comprehensively assess the amount of nutrients and foods consumed [12] and is less likely to be developed with the intention of measuring FV variety. In addition, because most of the time frames in studies that have measured FV variety using 24-h recall are 1–2 days, habitual understanding is limited.

In Japan, the strategic importance of assessing vegetable variety is higher than that of assessing fruit variety because more than 50% of those aged 20–49 years were found to have an intake of 0 g/day of fruit [13]. Although some studies have examined the characteristics of the increasing trend in the number of different foods consumed according to multiday dietary records [14,15], there is limited information about the number of different vegetables consumed. By estimating the capture proportion for each survey day and the strength of the correlation between survey days for the number of vegetables consumed, the validity of each survey day can be examined. Therefore, the aim of this study was to examine the number of survey days required to estimate habitual vegetable variety by conducting a multiday dietary record.

## 2. Materials and Methods

### 2.1. Participants

A cross-sectional study using an anonymous self-administered questionnaire was conducted in September 2018. The setting for this study was the following three communities in Japan: residents of Village A in a mountainous region of Niigata Prefecture (rural residents), students belonging to a general university in City B in Niigata Prefecture (general students), and fourth-year students belonging to a university for training registered dietitians in Town C in Nara Prefecture (nutrition students). According to the Census of Agriculture and Forestry [16], the forest area per total land is 62.0% in Village A, 7.5% in City B, and 1.4% in Town C. The total area of cultivated land under management per non-forest area is 32.3% in Village A, 44.0% in City B, and 18.5% in Town C.

The eligibility criteria for participants were adults aged 20 years or older who resided within the prefecture of the setting area. The population of participants consisted of approximately 400 rural residents in Village A, 4000 general students in City B, and 40 nutrition students in Town C. Rural residents were recruited through the residents’ association, general students were recruited through the students who were doing fieldwork in Village A, and nutrition students were directly recruited by the research staff. The sample size was set at approximately 20 participants for each community because in a previous study that evaluated food variety, each group had 24 participants [14]; in addition, the number of participants was limited to 20 after consultation with representatives of rural residents. A total of 67 questionnaires were distributed directly to participants, and 63 were collected by mail (response rate: 94.0%).

When the questionnaires were distributed, a request for the participants’ cooperation was enclosed; this request described the purpose and methods of this study and ethical considerations. The participants provided their informed consent by submitting the questionnaire. This study was conducted after review and approval by the Research Ethics Committee of Kio University of the last author’s previous institution (Approval No. H30-31).

### 2.2. Measures

Qualitative dietary records were maintained for seven consecutive days. Although a larger number of survey days is better for habitual understanding, more survey days leads to a lower rate of participant cooperation. Because it has been suggested that one-week dietary records (seven consecutive days) are best treated statistically as a single measure [17], the number of consecutive days was set at seven in this study. The recorded items were meal start time, name of the dish, name of the food, and information about the commercial products (product name, seller name, and store name). Regarding the time of recording, responses were recorded during the meal or after the meal (with participants either taking photos or forgetting to take photos). Although it is better to record during the meal, if participants completed the dietary record after the meal, they were asked to take photos for their own confirmation, which helped them to reduce food omissions. Since the purpose of this study was to investigate vegetable variety, water and tea were not recorded to reduce participant burden.

In principle, vegetable items were extracted from the items recorded by the participants on the dietary record form. If only the name of the dish was recorded (e.g., curry (Japanese curry and rice) or gyōza (pan-fried dumplings)), foods with a high frequency of use in high-rank web search recipes were adopted. If there was a record of the product or restaurant name that was purchased or consumed along with the dish name, the actual foods were identified by web search. For vegetable juice drinks, the vegetable with the highest content was extracted when the vegetable juice contained 10% or more. Vegetable condiments (e.g., tomato ketchup and ginger paste) were classified in the food group “seasonings and spices,” which includes foods with the same name [18], and were not counted as vegetable items. Vegetable seasonings in cup noodles were classified in the food group “cereals,” which includes instant foods with included condiments [18], and were also not counted as vegetable items. The vegetable items were extracted and confirmed by two students belonging to a university for training registered dietitians and then reconfirmed by one registered dietitian to increase the validity.

Vegetable variety was defined as the number of different vegetables consumed extracted by the above method. Vegetables were extracted by referring to the Standards Tables of Food Composition in Japan-2015-(Seventh Revised Edition) [18]. For the extraction, vegetables with the same classification but with different food names were defined as different items, while the same vegetables with different cooking and processing methods were defined as the same item. For example, in the case of vegetables classified as peas, pea sprouts and snow peas were counted as different items, while boiled and frozen green peas were counted as the same item (Table 1). Vegetables with different food names but in the same category and differing only in processing method (e.g., “daikon,” “kiriboshi-daikon,” and “pickles”) were counted as the same item.

The following sociodemographic characteristics of the participants were collected: age, gender, height, weight, and number of people living in the same residence. Height (m) and weight (kg) were self-reported and used to calculate body mass index (kg/m^2^). In addition, the amount of vegetable consumption has been found to be strongly correlated with vegetable variety in several studies [19,20,21]. Therefore, the questionnaire also asked for the habitual consumption of the number of vegetable servings [22], which was correlated with the amount of vegetable consumption assessed by the diet history questionnaire. The number of vegetable servings per day was answered on a 5-point ordinal scale from “Very few” to “≥7 servings.” The size of a vegetable serving was defined as approximately 70 g salad or boiled greens and was explained using photo images.

### 2.3. Data Analysis

Differences in sociodemographic characteristics, and the number of vegetable servings per day between groups were examined using one-way analysis of variance for continuous data (age and body mass index), the chi-square test for categorical data (gender and living alone), and the Kruskal-Wallis test for ordinal data (number of vegetable servings). The number of vegetables consumed was determined to be normally distributed by histogram and a normal probability plot. Therefore, differences in vegetable variety on seven consecutive days between groups were examined using repeated measures analysis of variance. The capture proportion of the number of vegetables consumed per survey day based on seven consecutive days was compared using one-way analysis of variance. A Bonferroni correction for multiple comparisons was applied for these individual comparisons. Vegetable variety by the number of survey days was compared by Pearson’s correlation coefficient.

Furthermore, the maximum theoretical number of vegetables consumed was calculated based on the method of Asato et al. [15], which examined the number increase in consumed food items. First, the authors took the reciprocal of the number of survey days on the horizontal axis and the reciprocal of the mean number of vegetables consumed in each group on the vertical axis and created an approximately straight line from each plot. Then, the maximum theoretical number was determined by the reciprocal of the intercept on the vertical axis. Although the estimation of such double reciprocal plots is considered unreliable [23], since the maximum theoretical number can be estimated, they were presented as reference values.

IBM SPSS Statistics 27 (IBM Japan, Ltd., Tokyo, Japan) was used for all statistical analyses. The level of significance was set at *p* < 0.05 (two-sided test).

## 3. Results

Of the 63 respondents, three were excluded because they did not provide sociodemographic information or the names of foods. Thus, a total of 60 participants (16 rural residents, 17 general students, and 27 nutrition students) who were assessed for all seven days were included in the analysis. The sociodemographic characteristics of the participants are shown in Table 2. In terms of the groups with the highest means and proportions for the sociodemographic characteristics, rural residents had the highest mean age, at 59.4 years old; nutrition students had the highest proportion of women, at 81.5%; rural residents had the highest mean body mass index, at 23.1 kg/m^2^; and general students had the highest proportion of those living alone, at 76.5% (all *p* < 0.05). The number of vegetable servings per day was significantly different among the groups (*p* = 0.015), and the number of servings was higher in rural residents than in general and nutrition students.

The vegetable variety of the participants is shown in Table 3. The vegetable variety for all participants showed a converging trend in the mean (variation) number of different vegetables consumed per number of days: 6.0 items for one day, 9.2 (+3.2) items for two days, 11.8 (+2.6) items for three days, 13.8 (+2.0) items for four days, 15.3 (+1.4) items for five days, 16.7 (+1.4) items for six days, and 17.9 (+1.2) items for seven days. Since there was no interaction effect between time and group, the main effect of group was analyzed independently. The number of vegetables consumed over seven consecutive days was significantly different in repeated measures (*p* = 0.013), and there were more items for rural residents than for general students and nutrition students in multiple comparisons. There were no significant differences between the groups in the capture proportion of the number of vegetables consumed per survey day based on seven consecutive days. Pearson’s correlation coefficient for the correlation with the number of vegetables consumed on seven consecutive days was not statistically significant only on the first day for nutrition students (r = 0.21, *p* = 0.292) and was strong after the third day for all groups (r > 0.70, *p* < 0.001).

To calculate the reference values, when the reciprocal of the number of survey days was taken on the horizontal axis and the reciprocal of the mean number of vegetables consumed in each group on the vertical axis, a high correlation was confirmed between them for each group (Pearson’s correlation coefficient, all r > 0.99, *p* < 0.001). The maximum theoretical number of vegetables consumed was 24.8 items for all participants, 27.0 items for rural residents, 24.8 items for general students, and 24.0 items for nutrition students. The capture proportion of the number of vegetables consumed for seven consecutive days calculated from the maximum theoretical number was 71.9% for all participants, 76.3% for rural residents, 69.4% for general students and 69.2% for nutrition students.

## 4. Discussion

Vegetable variety based on dietary records for seven consecutive days confirmed the differences between groups by repeated measurements. In this study, there was no significant group difference in the capture proportion per survey day based on seven consecutive days, regardless of the number of vegetables consumed between the groups. Furthermore, there was a significant correlation between the number of vegetables consumed on seven consecutive days and that consumed on two or more days in all groups (r > 0.50), which was especially strong on three or more days (r > 0.70). A few previous studies have used a 24-h recall over two days to determine vegetable variety [24,25]. The present study also suggested that a dietary survey of two or more days would provide an estimate of habitual vegetable variety. Incidentally, because significant correlations between the number of vegetables consumed over seven consecutive days and that consumed on two or more days were noted for participants who included weekends and only weekdays in the first two days, respectively (data not shown), the difference between weekdays and weekends was considered minimal.

Comparison between groups showed that vegetable variety over seven consecutive days was significantly higher in rural residents than in nutrition students. Some previous studies have reported that vegetable variety is associated with household income [26], education [27], social class [27], home ownership [27], and marital status [28]. One of the characteristics of the rural residents compared to students in this study was the low proportion of individuals living alone. Because it has been suggested that family or shared meal frequency is also associated with healthier dietary outcomes [29], the association between living with others and vegetable variety may be important. Furthermore, rural residents had the highest Pearson’s correlation coefficient between one and seven days in the present study, suggesting that regular eating habits may be related to increased vegetable variety. Although detailed analysis could not be conducted due to the limited number of participants in this study, the differences between groups may be influenced by each confounding factor. Therefore, future analysis based on factors related to vegetable variety is necessary. Furthermore, as previously reported [19,20,21], the group with higher vegetable consumption had a greater vegetable variety. Although the vegetable items were adopted from the food numbers in the Standards Tables of Food Composition in Japan-2015-(Seventh Revised Edition) [18], the Guidelines for Measuring Household and Individual Dietary Diversity by the Food and Agriculture Organization of the United Nations (FAO) [30] classify vegetable groups differently into categories such as vitamin A-rich vegetables and tubers, dark green leafy vegetables, and other vegetables. Because dietary diversity scores need to be adapted according to local contexts [30], it is also necessary to consider the habitual consumption of vegetable group variety in Japan.

### Limitation

This study had several limitations. The first limitation was that the survey was conducted only at a single point in time. According to the FAO guidelines [30], dietary diversity should be measured during different seasons for a more complete assessment of usual diet in rural communities. Since it has been suggested that there are seasonal differences in the amount of vegetable consumption [31], the capture proportion of the number of vegetables consumed per survey day may also be affected by season. Second, the number of survey days was not large enough. Because the capture proportion of the number of vegetables consumed for seven consecutive days calculated from the maximum theoretical number was approximately 70%, the habitual understanding was considered achieved. However, it should be noted that the limited number of survey days requires a large extrapolation, which may have resulted in errors in the maximum theoretical number. Third, because participants were recruited indirectly in several areas, the cooperation rate is unclear. Depending on the cooperation rate, the participants may have been more health conscious than the population. Fourth, if the participant did not provide the food name of the dish, vegetable items were selected from common recipes on the web. Therefore, some of the vegetable items may have been different from what the participants actually ate. There are limitations in understanding the consumption of commercial products because 33.6% of adults eat out at least once a week, and 45.6% of adults eat take-out lunches or prepared foods at least once a week according to the 2019 National Health and Nutrition Survey, Japan [13]. However, generalization may be accepted because vegetable items with a high frequency of use were adopted. Finally, the survey used qualitative dietary records. Because the amount of consumption was not investigated, no cut-off point has been established, and even the consumption of a small amount was categorized as one item. In a previous study that counted the items consumed in separate mixed dishes as main components [32], an ingredient in mixed dishes was assigned if it contributed at least 10% of the dish’s total weight or was listed among the top five components. Counting the number of vegetables consumed considering weight is needed in future studies.

## 5. Conclusions

Based on seven consecutive days, there were no significant group differences in the capture proportion of vegetables consumed on each survey day, and all groups showed a significant correlation with the number of vegetables consumed on two or more days. The present results suggested that a dietary survey of two or more days provides an estimate of habitual vegetable variety. Considering the habitual understanding of grouped vegetable variety is also needed in future studies.

## Figures and Tables

**Table 1:** Example of extraction of the number of vegetables consumed.

Item No. ^1^	Food and Description ^1^	Ex. 1	Ex. 2	Ex. 3	Ex. 4
	(Peas)				
	Pea sprouts				
06019	Stem and leaves, raw				
06329	Sprouts, raw				
06330	Sprouts, boiled	v			
06331	Sprouts, sautéed				
	Snow peas				
06020	Immature pods, raw				
06021	Immature pods, boiled	v			
	Snap peas				
06022	Immature pods, raw				
	Green peas				
06023	Raw				
06024	Boiled		v		
06025	Frozen		v		
06026	Canned in brine				
	(Japanese radish, Daikon)				
	Daikon, sprouts				
06128	Sprouts, raw			v	
	Daikon, cultivar for leaf use				
06129	Leaves, raw				
	Daikon				
06130	Leaves, raw				
06131	Leaves, boiled			v	
06132	Root with skin, raw				v
06133	Root with skin, boiled				
06134	Root without skin, raw				
06135	Root without skin, boiled				
	*Kiriboshi-daikon* ^2^				
06136	Raw				
06334	Rehydrated and boiled				v
06335	Rehydrated and sautéed				
	Pickles				
06137	*Nukamiso-zuke* ^3^				v
06138	*Takuan-zuke* ^4^				
	**Total Items**	**2**	**1**	**2**	**1**

^1^ Standards Tables of Food Composition in Japan-2015-(Seventh Revised Edition) [18], ^2^ Cut and dried daikon root, ^3^ Pickled in salty rice bran paste, ^4^ Pickled with rice bran and salt, made of salted daikon.

**Table 2:** Sociodemographic characteristics of the participants (*n* = 60).

	Total (*n* = 60)	Rural Residents (*n* = 16)	General Students (*n* = 17)	Nutrition Students (*n* = 27)	*p* ^1^
Age (year)	31.3 (18.6)	59.4 (14.7) ^a^	20.2 (0.8) ^b^	21.7 (0.7) ^b^	<0.001
Women (*n*)	35 (58.3)	8 (50.0)	5 (29.4) ^a^	22 (81.5) ^b^	0.002
BMI (kg/m^2^)	21.5 (2.5)	23.1 (2.8) ^a^	22.5 (2.0) ^a^	19.9 (1.7) ^b^	<0.001
Living alone (*n*)	26 (43.3)	0 (0.0) ^a^	13 (76.5) ^b^	13 (48.1) ^b^	<0.001
No. of V servings/day		_a_	_b_	_b_	0.015
Very few (*n*)	4 (6.7)	0 (0.0)	2 (11.8)	2 (7.4)	
1–2 servings (*n*)	32 (53.3)	5 (31.3)	10 (58.8)	17 (63.0)	
3–4 servings (*n*)	21 (35.0)	9 (56.3)	4 (23.5)	8 (29.6)	
5–6 servings (*n*)	2 (3.3)	1 (6.3)	1 (5.9)	0 (0.0)	
≥7 servings (*n*)	1 (1.7)	1 (6.3)	0 (0.0)	0 (0.0)	

BMI, body mass index; V, vegetable. Values are mean (standard deviation) or n (%). ^1^ Analysis of variance for age and BMI, chi-square test for gender and living alone, and Kruskal-Wallis test for number of vegetable servings (different letters in the same row indicate significant differences by Bonferroni correction for multiple comparisons).

**Table 3:** Vegetable variety of the participants (*n* = 60).

	Total (*n* = 60)	Rural Residents (*n* = 16)	General Students (*n* = 17)	Nutrition Students (*n* = 27)	*p* ^1^
No. of V consumed		_a_	_b_	_b_	0.013
1 days	6.0 (3.4)	7.9 (3.9)	5.3 (3.9)	5.4 (2.4)	
2 days	9.2 (4.3)	12.1 (3.7)	8.4 (4.1)	8.0 (4.0)	
3 days	11.8 (4.5)	14.4 (4.5)	10.5 (4.0)	11.1 (4.3)	
4 days	13.8 (4.5)	16.5 (4.5)	12.7 (4.2)	12.9 (4.2)	
5 days	15.3 (5.0)	18.3 (5.6)	14.1 (4.7)	14.2 (4.2)	
6 days	16.7 (5.4)	19.5 (5.7)	15.9 (5.8)	15.4 (4.3)	
7 days	17.9 (5.4)	20.6 (5.9)	17.2 (5.6)	16.6 (4.5)	
(reference value)					
Max theoretical No.	24.8	27.0	24.8	24.0	
Capture proportion					
1 days/7 days	33.5 (15.6)	37.2 (12.7)	29.2 (18.2)	34.0 (15.3)	0.334
2 days/7 days	51.3 (17.1)	59.3 (10.9)	48.0 (17.5)	48.6 (18.8)	0.091
3 days/7 days	66.0 (13.9)	70.2 (11.2)	61.8 (14.0)	66.2 (14.8)	0.221
4 days/7 days	77.5 (11.5)	80.8 (9.1)	74.7 (11.8)	77.4 (12.4)	0.315
5 days/7 days	85.6 (9.6)	88.5 (6.8)	82.4 (11.3)	85.9 (9.7)	0.190
6 days/7 days	93.1 (7.3)	94.6 (6.1)	92.0 (9.3)	93.0 (6.6)	0.609
(reference value)					
7 days/Max days ^2^	71.9	76.3	69.4	69.2	
Correlation coefficient					
1 days–7 days	0.62 ***	0.78 ***	0.69 **	0.21	
2 days–7 days	0.72 ***	0.89 ***	0.72 **	0.51 **	
3 days–7 days	0.84 ***	0.93 ***	0.79 ***	0.78 ***	
4 days–7 days	0.91 ***	0.95 ***	0.91 ***	0.87 ***	
5 days–7 days	0.94 ***	0.98 ***	0.90 ***	0.92 ***	
6 days–7 days	0.98 ***	0.98 ***	0.98 ***	0.97 ***	

V, vegetables. Values are the mean (standard deviation), reference value, or Pearson’s correlation coefficient (** *p* < 0.01, *** *p* < 0.001). ^1^ Repeated measures analysis of variance for the number of vegetables consumed among groups (different letters in the same row indicate significant differences by Bonferroni correction for multiple comparisons). ^2^ Maximum theoretical number of days.

## Data Availability

The datasets generated and analyzed during the current study are not publicly available due to privacy and ethical restrictions but are available from the corresponding author on reasonable request.
